# A Dual Approach of an Oil–Membrane Composite and Boron-Doped Diamond Electrode to Mitigate Biofluid Interferences

**DOI:** 10.3390/s21238063

**Published:** 2021-12-02

**Authors:** Madeleine DeBrosse, Yuchan Yuan, Michael Brothers, Aleksandar Karajic, Jeroen van Duren, Steve Kim, Saber Hussain, Jason Heikenfeld

**Affiliations:** 1 Novel Device Lab., University of Cincinnati, Cincinnati, OH 45221, USA; debrosmc@mail.uc.edu (M.D.); yuanyh@mail.uc.edu (Y.Y.); karajiar@ucmail.uc.edu (A.K.); 2 711th Human Performance Wing, Air Force Research Laboratory, Wright-Patterson AFB, Dayton, OH 45433, USA; michael.brothers.4.ctr@us.af.mil (M.B.); steve.kim.13@us.af.mil (S.K.); saber.hussain@us.af.mil (S.H.); 3 Diamond Foundry Inc., South San Francisco, CA 94080, USA; jeroen@diamondfoundry.com

**Keywords:** biosensors, membranes, diamond, redox, interferents, foulants

## Abstract

Electrochemical biosensors promise a simple method to measure analytes for both point-of-care diagnostics and continuous, wearable biomarker monitors. In a liquid environment, detecting the analyte of interest must compete with other solutes that impact the background current, such as redox-active molecules, conductivity changes in the biofluid, water electrolysis, and electrode fouling. Multiple methods exist to overcome a few of these challenges, but not a comprehensive solution. Presented here is a combined boron-doped diamond electrode and oil–membrane protection approach that broadly mitigates the impact of biofluid interferents without a biorecognition element. The oil–membrane blocks the majority of interferents in biofluids that are hydrophilic while permitting passage of important hydrophobic analytes such as hormones and drugs. The boron-doped diamond then suppresses water electrolysis current and maintains peak electrochemical performance due to the foulant-mitigation benefits of the oil–membrane protection. Results show up to a 365-fold reduction in detection limits using the boron-doped diamond electrode material alone compared with traditional gold in the buffer. Combining the boron-doped diamond material with the oil–membrane protection scheme maintained these detection limits while exposed to human serum for 18 h.

## 1. Introduction

Significant progress has been made towards establishing electrochemical biosensors in health-monitoring systems, yet only a few biosensors other than glucose monitors have found significant commercial application [1]. Most analytes outside of glucose and alike metabolites, such as proteins and hormones, maintain much lower biological concentrations than is detectable by electrochemical biosensors in their current state [2,3,4]. A key issue for the detection of low concentration analytes is the background signal present from both water electrolysis as well as other redox-active solutes, such as NADH and FADH_2_, inherently present in biofluids [5,6,7]. Furthermore, fouling in real biofluids also changes the voltage drop at the electrode surface, thus reducing the observed current and its apparent biosensor signal. Even perfectly selective probes, such as enzymes, do not solve this issue, where background interference current limits operation to analyte concentrations in the μM to mM range (or higher). This, in part, explains the limitation of enzymatic sensors to a subset of high concentration metabolites [6,7]. 

**Figure 1:**
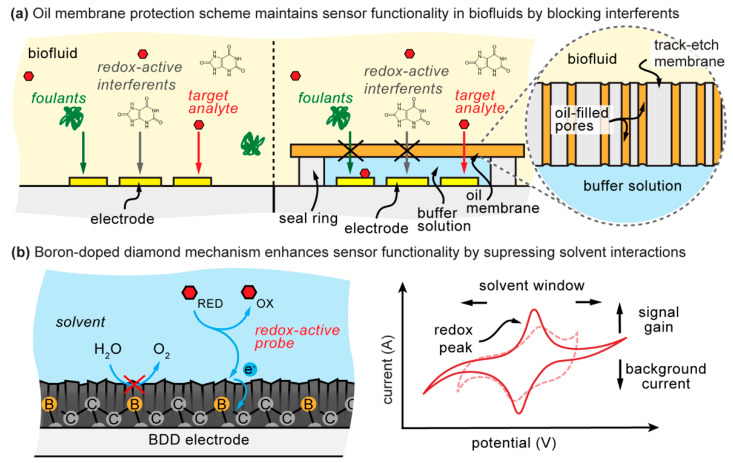
Combined oil–membrane protection and boron-doped diamond (BDD) approach for mitigating biofluid effects. (**a**) The oil–membrane protection scheme encapsulates the sensor within its ideal buffer environment separate from the biofluid, simultaneously blocking unwanted hydrophilic interferents (i.e., foulants, redox-active species) and partitioning hydrophobic analytes. (**b**) Boron-doped diamond reduces solvent interactions by suppressing kinetically slow inner-sphere, solvent-based electron transfer while facilitating comparatively faster analyte electron transfer, thereby increasing sensor functionality in fluid environments. As a result, boron-doped diamond displays a signal gain increase, background current reduction, and solvent window broadening (solid red line) compared to traditional electrode materials (dotted red line).

Due to all these challenges, there exists a fundamental gap between biosensors and their application for real-world use. However, if background interference current from solutes and water can be minimized, even nanomolar detection could be possible with the existing enzymatic detection approaches. More commonly, however, research is focused on tedious probe development, which frequently limits its benefits to a few select target analytes. Ideally, any such solution that resolves background interference would be highly generalizable (generically working against the solvent and most of its solutes). Herein we provide methods to solve both—solute and solvent interactions—and lower the detection limits for established biosensors by shifting the research focus to versatile surface chemistries. We present a combined boron-doped diamond electrode and oil–membrane protection approach (Figure 1) that encapsulates the biosensor in its ideal environment, forming a semipermeable barrier to mitigate the matrix effects of the surrounding biofluid. Simply stated, the boron-doped diamond electrode surface suppresses the background interference current due to water electrolysis. At the same time, the oil–membrane interface blocks the majority of interferents in the blood that are hydrophilic and unable to permeate easily through the hydrophobic oil–membrane [8,9]. Simultaneously, hydrophobic analytes may pass through the oil–membrane to the inner biosensor region. Analyte permeability depends primarily on oil–water partitioning properties, as defined by the partition coefficient *K*. Under ideal conditions, the diffusing species will possess a positive (hydrophobic) *K*-value so as to energetically favor diffusing into the oil–membrane and possess a low—but not negative—*K*-value so that it diffuses back into the aqueous biosensor region. As a result, analyte detection is limited to semihydrophobic analytes that can traverse the oil–membrane, such as steroid hormones and orally administered small-molecule drugs, which themselves represent large sets of clinically relevant analytes in blood [10]. Our results demonstrate > 300-fold reduction in the limit of electrochemical detection in human serum without a biorecognition element but also reveal a challenge in boron-doped diamond electrode reproducibility. With further optimization, boron-doped diamond and oil–membrane protection should enable enzymatic and other electrochemical sensors with significantly lower detection limits in raw biofluids.

## 2. Background and Limitations of Boron-Doped Diamond Electrodes

Solvent and solute effects are inherent challenges to biosensing in raw biofluids [5,6,7]. These effects cause an increase in the background current, thereby increasing the limit of detection (LOD) of the analyte of interest. Gold-based electrodes have been the benchmark for biosensor development as their surface chemistry allows for facile binding of a variety of sensing moieties while simultaneously possessing both conductivity and chemical stability [11,12]. However, these same properties that make gold a versatile biosensor electrode material also allow for random absorption of unwanted species present in all biofluids. In addition, gold exhibits a strong electrolysis current of water. Water electrolysis current benefits from the proximity of multiple water molecules covalently bound at electrically active sites for water oxidation–reduction reaction to occur; this is inherent in most commonly used electrodes. In addition to water, there are numerous redox-active background interferents in the blood that also negatively impact the limit of detection [13,14].

Carbon-based electrodes have become increasingly popular due to their resistance to surface binding, their wide potential windows for measurement, and broad versatility. Standing out amongst these electrodes is the conductive boron-doped diamond, boasting the broadest potential window of any known material. Boron-doped diamond exhibits high chemical stability and resistance to surface binding, making it an ideal candidate for biosensor applications [15,16]. These resistant properties arise from its inert crystalline diamond structure, which is then doped with boron impurities to induce metal-like conductivity [17]. As boron-doped diamond substrates are grown from the bottom up, their properties—dopant concentration, sp^2^ content, and surface termination—can be tuned for specific applications. For example, increasing nondiamond-carbon sp^2^ content at the surface greatly enhances electrocatalytic activity compared with the far more inert diamond sp^3^ hybridization. This enables electrochemical detection of redox-active species previously insensitive to detection, such as dissolved oxygen and carbon dioxide in arterial blood samples of hospitalized patients [18,19,20]. However, an increase in electrocatalytic activity also enhances electron transference, including solvent and solute interactions, thereby narrowing the potential window of measurement and increasing background current [17]. Therefore, boron-doped diamond properties can be tuned so that solvent-specific inner-sphere interactions are minimized while maintaining the electrocatalytic activity of the target analyte. 

The current state-of-the-art work for boron-doped diamond functionality in biofluids remains contradictory. Literature has shown boron-doped diamond displays significant improvement in detection limits of a variety of analytes and appears to be unaffected by the presence of biofluid, maintaining sensor performance for up to several hours [21,22]. However, others have shown that boron-doped diamond is not immune to the performance-reducing effects of biological fluids [23,24,25]. Conventional methods of mitigating these effects involve the covalent binding of a protective polymer layer. Poly(ethylene glycol) (PEG) has often been referred to as the standard antifouling polymer, but its application to boron-doped diamond surfaces is much more intensive compared to self-assembling monolayer (SAM) on gold surfaces [26,27]. The immobilization of molecules to boron-doped diamond generally requires an amine-terminated surface and reaction with a crosslinker. [28]. Others have taken advantage of “click” chemistry by modifying the boron-doped diamond surface with either an alkyne or azide termination to rapidly adjoin biomolecules to the surface [29]. A significant disadvantage of these methodologies is the dependence on surface functionalization, thereby altering the potentially advantageous properties of boron-doped diamond, and therefore the detection limits.

In this work, instead of relying on the boron-doped diamond electrode for all the desired functionality in a biosensor placed in blood, we allow the boron-doped diamond to remain as a bare surface for suppression of water electrolysis current while relying on a protective oil–membrane to block both foulants and most redox-active solutes found in the blood. For these reasons, the results will successfully show a decrease in detection limit by >300 times even in human serum.

## 3. Materials and Methods

### 3.1. Reagents and Materials

All reagents, oils, and human serum (from male AB clotted whole blood) were purchased from Sigma-Aldrich (St. Louis, MO, USA). The polycarbonate track-etch (PCTE) membranes (1 μm diameter pore size, PVP-free) were purchased from Sterlitech Corporation (Kent, WA, USA). Samples of Kapton^®^ polyimide single-sided tape and Brampton marine epoxy were purchased from Amazon (Seattle, CA, USA). The 1.25 mm thick acrylic electrode backing was purchased from McMaster-Carr (Princeton, NJ, USA). FluoroPel Plastics copolymer solution was purchased from CYTONIX (Beltsville, MA, USA). Gold rod working (2 mm gold diameter), silver/silver chloride reference, and platinum counter electrodes were purchased from CH Instruments (Austin, TX, USA). Fisher Scientific 4M potassium chloride saturated with silver chloride was used as the reference electrode filling solution (Waltham, MA, USA). Conductive O-terminated boron-doped polycrystalline diamond films on conductive silicon were obtained from Diamond Foundry (San Francisco, CA, USA). An electrode polishing kit was purchased from eDAQ (Colorado Springs, CO, USA). All experiments were conducted in ultrapure water (resistivity = 18.2 MΩ·cm), and titration solutions were prepared fresh daily.

### 3.2. Apparatus

Electrochemical experiments, including cyclic voltammetry (CV) and two-step chronocoulometry (CC), were performed on a CH Instruments Models 600E Series Potentiostat/Galvanostat (Austin, TX, USA). A Four Dimensions Six-Point-Probe Meter 101C was used to measure sheet resistivity of the boron-doped diamond surface. Laser-cut materials were crafted using a Universal Laser System VLS3.50 (Scottsdale, AZ, USA), and 3D-printed materials were assembled on a FormLabs Form 2 (Somerville, MA, USA) using FLGPCL02 clear resin.

### 3.3. Electrode Storage and Preparation

Gold rod electrodes were stored in ambient air at room temperature. These electrodes were first physically cleaned using both 0.30 µm and 0.05 µm alumina slurries on polishing pads, according to manufacturer instructions, for one minute each. Next, the electrodes were electrochemically cleaned via cycling in 0.5 M NaOH (−1.0 to −1.6 V, 120 scans) and 0.5 M H_2_SO_4_ (0 to 1.6 V, 120 scans) successively.

Boron-doped diamond electrodes were fabricated by first applying a layer of gold on one edge of a piece of the boron-doped diamond-coated conductive silicon wafer. To create a well-defined electrode area, the boron-doped diamond material surface was then insulated by carefully pressing a section of single-sided Kapton^®^ polyimide tape containing a 2-mm diameter opening for measurement, leaving the gold-sputtered portion uncovered on the opposite end of the opening. The insulated boron-doped diamond material was adhered to a 1.25-mm acrylic backing, and its exposed sides were covered using marine epoxy. A labeled photograph of the final electrode is provided with the online supplementary material (Figure S1). The boron-doped diamond material and electrodes were stored at room temperature and under nitrogen. Due to the delicate boron-doped diamond thin-film, these electrodes were exclusively electrochemically cleaned in 0.5 M H_2_SO_4_ under an extended potential range (0 to 2.2 V, 120 scans) as recommended for oxygen-terminated boron-doped diamond materials [30]. Longer cleaning cycles were not performed as the marine epoxy used to secure the electrode components softened in the acidic environment. Boron-doped diamond material was stored under nitrogen at room temperature.

### 3.4. Electrochemical Measurements

Initial CV curves were performed (100 mV/s scan rate) to determine the potential window of the electrode material, background current, and suitable redox potentials of the redox-active probes (potassium hexacyanoferrate (II/III) and hexaammineruthenium (II/III) chloride). Signal change as a function of redox-active probe concentration was monitored using two-step chronocoulometry (30-s step). The applied redox potentials in potassium hexacyanoferrate (II/III) tests were −0.1 and 0.4 V for gold electrodes and −0.5 and 0.8 V for boron-doped diamond electrodes. The applied redox potentials in hexaammineruthenium (II/III) chloride tests were −0.1 and 0.4 V for both gold and boron-doped diamond electrodes. A three-electrode system—gold/boron-doped diamond working, platinum counter, and silver/silver chloride reference electrodes—was employed and placed in a laser-cut electrode mount to maintain electrode-spacing consistency between tests. All tests were performed individually using new or cleaned working electrodes in triplicate. Gold and boron-doped diamond electrodes underwent five baseline conditioning steps at their designated chronocoulometric potentials prior to the start of titration. The total number of scans applied to each new electrode did not exceed 15 as signal outcomes were increasingly affected by batch-to-batch variability with the boron-doped diamond material (Figures S2 and S3).

### 3.5. Oil–Membrane Composite Protection

A 3D-printed U-boat setup was utilized to investigate the protective performance of separating the boron-doped diamond electrode from the biofluid by an oil-impregnated track-etch membrane. A labeled photograph of the U-boat setup is provided with the online supplementary material (Figure S4). The U-boat was coated with FluoroPel 1601 V fluoropolymer to minimize nonspecific surface adsorption to or into the plastic. The membrane was soaked in the desired oil (no-oil or castor oil) and placed between the two fluid compartments of the U-boat. Each fluid compartment possessed an O-ring that held the membrane in place. The U-boat setup was secured by a bar clamp and examined for leaks. 1× PBS was placed in one chamber (buffer side), and human serum was placed in the opposite chamber (biofluid side). The setup was incubated for 18 h to allow adequate time for contents to diffuse and reach a general state of equilibrium. Following the incubation period, contents on the buffer side were titrated with the redox-active probe.

### 3.6. Analysis of Titration Data

The raw chronocoulometry data was exported from a saved text file from the CH Instrument (Austin, TX, USA) Software. The signal was calculated as a change in charge between 0 and 30 s of the oxidation step (∆Charge). ∆Charge values for each of the titration point concentrations were plotted in terms of the ‘∆Charge vs. Concentration’ of the redox-active probe. A linear trendline was applied to the linear region of the plotted data points (R^2^ > 0.99). The limit of detection was defined as 3∗σ/m, where *σ* is the standard deviation of the blank (*n* = 4) and *m* is the slope of the linear trendline. Electrode sensitivity was defined as the trendline slope. Each experiment included four independent trials (*n* = 4). As this is a comparative study, a more rigorous analytical analysis was not performed due to the high test-by-test variability. The limit of detection and sensitivity was expressed in terms of ranges to encompass all measurement fluctuations.

## 4. Results and Discussion

### 4.1. Boron-Doped Diamond Displays Wide Potential Window

**Figure 2:**
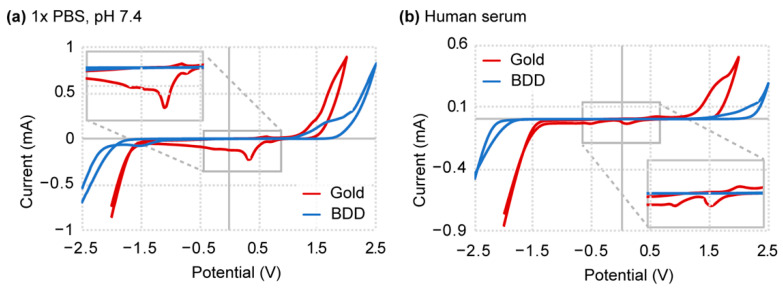
Cyclic voltammetry of boron-doped diamond (BDD) and gold electrodes in (**a**) 1× PBS and (**b**) human serum. Boron-doped diamond displayed extended solvent windows and reduced background in both fluids compared to gold.

Our first objective was to broadly assess the electrocatalytic properties of our specific boron-doped diamond sample and get a sense of how its reactivity compares to expected boron-doped diamond values. Unmodified standard gold electrodes were used as a means of comparison, considering their well-characterized susceptibility to solvent and solute effects [31,32,33]. With regard to properties, we specifically focused on the span of the potential window and the background current density as it directly relates to these solvent and solute interactions. A cyclic voltammetry curve for boron-doped diamond and gold electrodes was generated in both buffer (1× PBS, pH 7.4) and human serum (Figure 2). A larger magnitude of the boron-doped diamond potential window is immediately apparent compared to the gold electrode in both buffer (1× PBS) and biofluid (human serum) environments (Figure 2). This observation remains in agreement with literature where the potential window of the boron-doped diamond often spans a range of 2 V or greater, depending on its composition [16]. Closer inspection of the current density shows boron-doped diamond at a consistently reduced background across the length of the window, suggesting low environmental interferences as well as depressed electrocatalytic activity compared to gold. Sources of these environmental interferences may arise from redox-active biomolecules known to exist in serum, such as NADH, FADH_2_, monoamine neurotransmitters, uric acid, and other redox-active analytes, which contribute to the measured current. In addition, the anodic and cathodic peaks inherently associated with gold in both cyclic voltammetry curves generate further interfering background current. We confirmed the semiconductive property of boron-doped diamond from sheet resistivity measurements (3 to 8 Ω-cm). Again, this information coincides with literature results as boron-doped diamond is innately more inert but maintains conductive properties [15,16]. Interestingly, both gold and boron-doped diamond materials show background current suppression when placed in human serum (Figure 2b). This current suppression suggests non-specific surface adsorption that impedes electron transfer kinetics, causing a decrease in current density and potential window broadening [34]. How these biofluid properties affect sensor performance are examined in the following sections.

### 4.2. Boron-Doped Diamond Outperforms Gold in Buffer

Our next objective was to evaluate boron-doped diamond electrode performance within an ideal environment. This provided us with benchmark values for comparison when the electrode is placed in a raw biofluid. In this case, our selected “ideal” environment was 1× PBS (pH 7.4) as it is a commonly used buffer in biological applications and an established matrix for biosensor characterization. Performance was assessed in terms of analytical characteristics, specifically the detection limit and sensitivity of the redox-active species hexacyanoferrate (II/III). We chose hexacyanoferrate (II/III) as it is an inner-sphere redox probe and is, therefore, more susceptible to surface composition and interferences. We intended to monitor surface electrode behavior in differing fluid environments; therefore, we elected a chronocoulometry method to measure the change in surface charge generated by the probe and monitor the effects of possible interferents and absorbed species.

**Figure 3:**
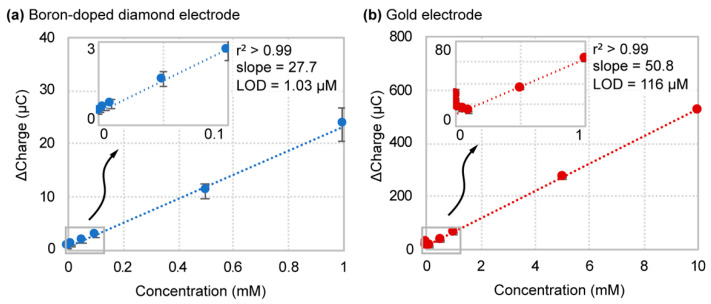
Hexacyanoferrate (II/III) titration and linear regression curves for (**a**) boron-doped diamond and (**b**) gold electrodes in 1× PBS.

Results obtained for titration of hexacyanoferrate (II/III) in 1× PBS are shown in Figure 3. Initially, we observed more significant variability in the measurements for boron-doped diamond than in gold. This makes sense since the boron-doped diamond substrate is not a pure crystalline structure, thus causing batch-to-batch variations in the dopant concentration and polycrystallinity between fabricated electrodes, and therefore variations in the electrocatalytic properties. This was confirmed by the observed fluctuations in resistivity measurements across the surface of the boron-doped diamond material. In connection with sensitivity, as defined by the slope of the linear regression curve, the gold electrode surpassed boron-doped diamond with a sensitivity of 50.8 ± 0.6 ∆C/mM and 22.7 ± 2.6 ∆C/mM, respectively. However, when titration results were plotted in terms of ‘Percent Signal Gain (%SG) vs. Concentration’, the defined sensitivity for boron-doped diamond was 3400 ± 1500 %SG/mM, over 40 times greater than that of the gold electrode (161 ± 30 %SG/mM) (Figure S5). The heightened sensitivity in terms of percent signal gain arises from the lower baseline signal of a boron-doped diamond. Higher concentrations of hexacyanoferrate (II/III) generated a signal plateau in a boron-doped diamond that we speculate is due to the saturation of conductive boron sites within the diamond structure. For this reason, higher redox probe titration concentrations for a boron-doped diamond in 1x PBS were not plotted. The limit of detection calculated from the plotted data shows boron-doped diamond at a LOD of 1.03 ± 0.43 µM, whereas gold displayed a much broader and elevated range of 116 ± 63 µM. This equates to an average 140-fold reduction in the detection limit and a 365-fold reduction between the highest gold and lowest boron-doped diamond electrode LOD values. Regarding biosensors applications, this level of detection limit reduction could mean that previously ineffective sensors systems restricted by analyte concentration barriers could now function at biologically relevant ranges. Additionally, the apparent boron-doped diamond saturation would not limit practical applications as most clinically relevant analyte biological reference ranges fall below the mM regime.

### 4.3. Boron-Doped Diamond Performance Significantly Reduced in Biofluid

**Figure 4:**
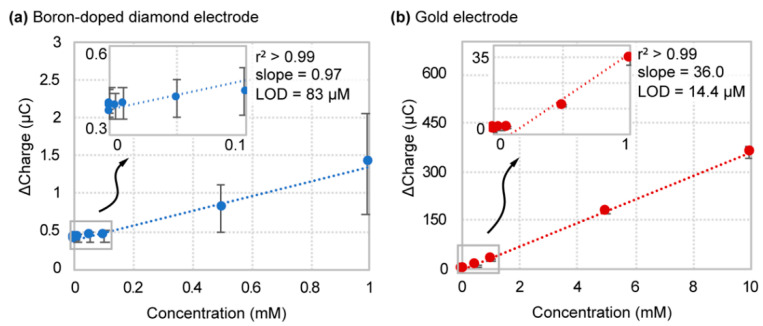
Hexacyanoferrate (II/III) titration and linear regression curves for (**a**) boron-doped diamond and (**b**) gold electrodes in human serum.

With an “ideal” benchmark established for boron-doped diamond, performance was next evaluated within a realistic biofluid environment. We chose human serum as it contains redox reactive interferents (e.g., NADH, acetic acid, uric acid, and creatine) as well as an abundance of biofouling proteins such as albumin. An identical titration and calculation procedure was used, as previously stated in the buffer studies. Obtained results for titration of hexacyanoferrate (II/III) redox probe in human serum are shown in Figure 4. A comparison of electrode analytical performance characteristics in both buffer and biofluid environments can be found in Table 1 at the end of this section.

Focusing first on the similarities, boron-doped diamond test-by-test variability carries over from the buffer titration data, while gold shows more consistent measurements. However, this is the only association that can be made between buffer and serum electrode performance. In fact, in human serum, the gold electrode material surpasses boron-doped diamond in both detection limit and sensitivity. Boron-doped diamond displayed a detection limit of 83 ± 47 µM versus 15.4 ± 3.8 µM for gold—a 6-fold reduction in detection limit on average. In addition, the sensitivity of boron-doped diamond and gold decreased to 0.97 ± 0.53 ∆C/mM and 36.0 ± 1.5 ∆C/mM, respectively, compared with buffer measurements. However, sensitivity in terms of signal gain increased for the gold electrode in human serum (1300 ± 280 %SG/mM) (Figure S6). This decrease in sensitivity of boron-doped diamond compared to gold suggests that this electrode material is more susceptible to surface fouling and favors fouling by larger solutes such as albumin. These results were further supported by cyclic voltammetry measurements of the redox probe in buffer and serum (Figure S7). The cyclic voltammogram for the gold electrode displayed significant peak current intensity reduction and peak potential broadening but maintained a visible current response and reversibility. In contrast, the boron-doped diamond electrode exhibited a sharp reduction in peak current density to near baseline levels. Since hexacyanoferrate (II/III) detection by boron-doped diamond requires surface interaction for electron transfer to occur, the presence of interfering species at its surface would be highly detrimental to sensor performance. For this reason, it is imperative that boron-doped diamond remains in an ideal fluid, protected from a raw biofluid environment. This need for protection directly motivated our use of the oil–membrane sensor encapsulation scheme of the boron-doped diamond electrode.

### 4.4. Oil–Membrane Protection Maintains Boron-Doped Diamond Performance in Biofluids

As previously shown in Figure 3, the electrode performance of boron-doped diamond outperforms gold in relation to detection limit by a 140-fold reduction under ideal conditions. However, as shown in Figure 4, these beneficial sensor properties are completely diminished in the presence of biofluid. In biofluids, the majority of the most problematic interfering solutes exhibit hydrophilic properties, including salts, acids, bases, and larger molecules such as proteins that must be hydrophilic to maintain their solubility in biological matrices. Generalizing the problematic solutes as hydrophilic theoretically presents a significant opportunity for filtering out these species by implementing a hydrophobic protective barrier for sensors systems. Here we implement an oil-impregnated polycarbonate track-etch (PCTE) membrane as a semipermeable hydrophobic filter to mitigate the diffusion of hydrophilic interfering species [35]. The oil–membrane conditions used during this study include no-oil as a control and castor oil as a robust hydrophobic barrier. Castor oil has been shown to maintain buffer conditions over a 12-h timespan, likely due to its high viscosity and high water-octanol partition coefficient that considerably slow the passage of hydrophilic species [35]. Other oil–membrane composites were not investigated as our goal was to simply demonstrate the ability of the oil–membrane in maintaining sensor performance in biological fluids. 

**Figure 5:**
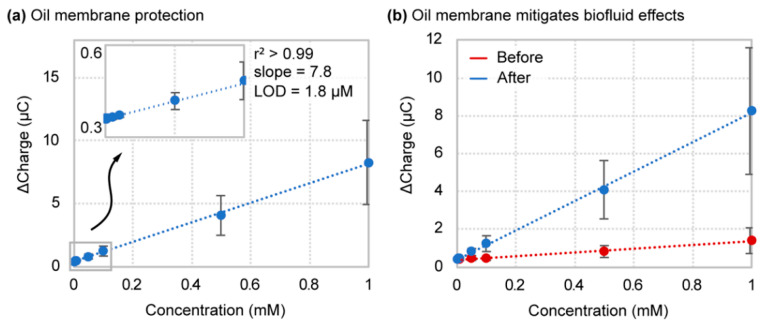
Hexacyanoferrate (II/III) titration and linear regression curves for (**a**) oil–membrane protected boron-doped diamond. (**b**) A before-and-after comparison of oil–membrane implementation for the boron-doped diamond electrode.

The analytical performance results for the boron-doped diamond electrode successfully validate the protection ability of the oil membrane from the negative effects of biofluid environments. The oil–membrane protection scheme maintains boron-doped diamond performance within a detection limit of 1.8 ± 1.3 µM (Figure 5a), which equates to an 84-fold average improvement and up to a 247-fold reduction under ideal circumstances, in detection limit compared with boron-doped diamond directly in human serum (Figure 5b). These analytical performance results, while not a complete restoration to “ideal” fluid values, nonetheless outperform the gold electrode based on detection limit. Electrode sensitivity also increased to 7.8 ± 2.8 ∆C/mM from 1.19 ± 0.45 ∆C/mM in human serum (Figure 5). Looking at sensitivity in terms of percent signal gain from baseline, we see a sensitivity range (1100 ± 340 %SG/mM), which approaches ideal buffer conditions (Figure S8a). Hexacyanoferrate (II/III) titration results for the membrane containing no oil confirm that membrane protection is dependent on the presence of the oil phase (Figure S9b). By removing the oil, electrode performance is reduced to raw biofluid levels (Figure S9a). It should be noted that the increase in the variability of the results upon implementation of the oil–membrane is understandable since the composition of the oil-impregnated membrane itself possesses inherent inconsistencies between replicate tests (i.e., oil layer thickness and consistency). These conditions could be improved by incorporating a membrane substrate that interacts more strongly with the oil phase so that the oil remains more reliably within its pores. Additionally, optimization of the membrane substrate and oil composition for its specific application would enhance its protective abilities up to the extent of ideal environment conditions. Such improvements would, in essence, shrink the gap between new biosensor discovery and application. These analytical performance results for the boron-doped diamond in conjunction with the oil–membrane protection scheme using hexacyanoferrate (II/III) are summarized in Table 2.

**Figure 6:**
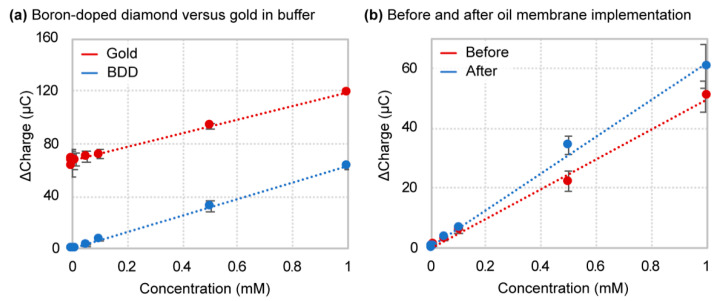
Hexaammineruthenium (II/III) titration and linear regression curves for (**a**) boron-doped diamond versus gold electrodes. (**b**) A before-and-after comparison of oil–membrane implementation for the boron-doped diamond electrode.

We have shown that the oil–membrane protection scheme combined with the boron-doped diamond electrode mitigates biofluid interferences and maintains its superior analytical performance using hexacyanoferrate (II/III). To further confirm these results, we also chose hexaammineruthenium (II/III) as a redox-active probe in our remaining performance assessments. Immediately noticeable in the titration results in Figure 6a is the positive shift of the gold electrode titration curve for hexaammineruthenium (II/III). This is likely due to the position of the hexaammineruthenium (II/III) redox potentials centered on −0.4 V to −0.1 V (vs. Ag/AgCl), directly within the region in which gold displays heightened background current (Figure 2a). The boron-doped diamond material suppresses these fluid interactions and therefore does not suffer from high background current interferences. As a result, the limit of detection of hexaammineruthenium (II/III) titration in buffer was 1.6 ± 1.2 µM for boron-doped diamond, compared to 155 ± 120 µM in gold. This difference equates to an average of 185-fold reduction in detection limit and up to over a 300-fold reduction under ideal conditions. Electrode sensitivity of boron-doped diamond and gold exhibit comparable values at 62.9 ± 3.2 ∆C/mM and 52.6 ± 3.9 ∆C/mM, respectively. The analytical performance results for hexaammineruthenium (II/III) in 1× PBS buffer are summarized in Table 3.

Upon placing the boron-doped diamond electrode in human serum, both analytical performance parameters were reduced, yielding a limit of detection of 6.7 ± 3.1 µM and a sensitivity of 48.9 ± 4.6 ∆C/mM—a far lesser magnitude compared to the inner-sphere redox probe. These results were confirmed in cyclic voltammetry curves of the redox probe, where peak potential and current intensity only differed slightly between buffer and serum measurements (Figure S10). This is likely due to the enhanced surface interactions of hexaammineruthenium (II/III) to boron-doped diamond, which limit the impact of biofluid interferences and fouling. Hexaammineruthenium (II/III) is not only a positively charged redox-active probe, but its amine groups bind sp^2^ carbon sites, both enhancing attractive interactions to the negatively charged O-terminated boron-doped diamond surface. In contrast, hexacyanoferrate (II/III) is a negatively charged redox-active probe and therefore experiences greater repulsive forces to the boron-doped diamond. Figure 6b shows a complete reversal of biofluid effects upon oil–membrane implementation, with a limit of detection of 7.3 ± 4.2 µM and a sensitivity of 61.5 ± 6.0 ∆C/mM. This equates to sensitivity in percent signal gain equal to 24,000 ± 3300 %SG/mM (Figure S9b). While the advantage of the oil–membrane protection scheme compared to biofluid under these circumstances is less striking than the hexacyanoferrate (II/III) titration results, it shows that this dual approach is applicable for multiple sensor systems. These analytical performance results for the boron-doped diamond in conjunction with the oil–membrane protection scheme using hexaammineruthenium (II/III) are summarized in Table 4.

## 5. Conclusions

In this work, we present a dual approach to mitigate both solute and solvent effects while simultaneously improving detection limits by over 100-fold compared to traditional electrodes by (1) employing boron-doped diamond material and (2) incorporating an oil–membrane protection scheme. From an application perspective, our approach is highly generalizable and presents an opportunity for current sensor systems to function in biofluids with fewer performance-diminishing interferent effects. Integrating such an approach into a fully realized device would not require changing the sensor system itself; therefore, highly specific recognition elements (i.e., enzyme- and aptamer-based) could readily leverage our demonstrated approach if they can be fabricated with a diamond-based electrode. 

We believe the most important application of the present work is to enable nM level detection limits with enzymatic sensors. Enzymatic sensors can take advantage of the oil–membrane hydrophobicity to enhance the detection range of target analytes and enable sensors that can monitor lower concentration analytes using more exotic enzymes. For example, in enzymatic sensors, the oil–membrane can be used to trap hydrophilic redox reporters within the sensor environment while enabling the hydrophobic target analyte to diffuse into or out of the device as needed. As the enzyme interacts with its target, converted redox reporters from the enzyme reactions can build up within the sensor compartment. In this way, we can trade time-resolution for sensitivity to improve the limit of detection. To move this and other works forward, the inherent variability of the boron-doped diamond material, as well as the oil–membrane technology, should be addressed but already shows promising results without optimization. 

## Figures and Tables

**Table 1:** Summary of analytical performance characteristics between boron-doped diamond and gold electrodes for hexacyanoferrate (II/III) titration.

Environment	Electrode	Sensitivity (ΔC/mM)	LOD (μM)	LOD Fold Reduction (High)
1× PBS	BDD	22.7 ± 2.6	1.03 ± 0.43	142 (365)
	Gold	50.8 ± 0.6	116 ± 63	
Serum	BDD	0.97 ± 0.53	83 ± 47	---
	Gold	36.0 ± 1.5	15.4 ± 3.8	

**Table 2:** Summary of the analytical performance of boron-doped diamond with and without integrated oil–membrane protection in human serum for hexacyanoferrate (II/III) titration.

Membrane	Sensitivity (ΔC/mM)	LOD (μM)	LOD Fold Reduction (High)
Castor Oil	7.8 ± 2.8	1.8 ± 1.3	84 (247)
No Oil	1.19 ± 0.45	82 ± 43	

**Table 3:** Summary of analytical performance characteristics between boron-doped diamond and gold electrodes for hexaammineruthenium (II/III) in 1× PBS.

Electrode	Sensitivity (ΔC/mM)	LOD (μM)	LOD Fold Reduction (High)
BDD	62.9 ± 3.2	1.6 ± 1.2	185 (303)
Gold	52.6 ± 3.9	155 ± 20	

**Table 4:** Summary of the analytical performance of boron-doped diamond with and without integrated oil–membrane protection in human serum for hexaammineruthenium (II/III) titration.

Membrane	Sensitivity (ΔC/mM)	LOD (μM)	LOD Fold Reduction (High)
Castor Oil	61.5 ± 6.0	7.3 ± 4.2	13 (34)
None	48.9 ± 4.6	6.7 ± 3.1	

## Data Availability

Not applicable.

## Supplementary Materials

The following are available online at https://www.mdpi.com/article/10.3390/s21238063/s1, Figures S1–S10.
