# Supplementary material for: A Dual Approach of an Oil–Membrane Composite and Boron-Doped Diamond Electrode to Mitigate Biofluid Interferences
Source: Sensors (Basel). 2021 Dec 2;21(23):8063. doi: 10.3390/s21238063 (PMC8659581; doi:10.3390/s21238063)
Supplement: Supplementary file 1 [file sensors-21-08063-s001.zip › sensors-1447089-supplementary.pdf]

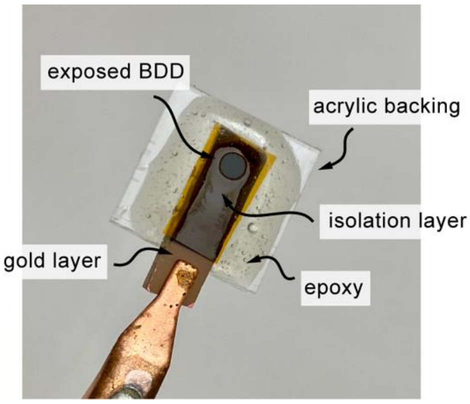

Figure S1. Labeled photograph of fully constructed boron-doped diamond electrode.

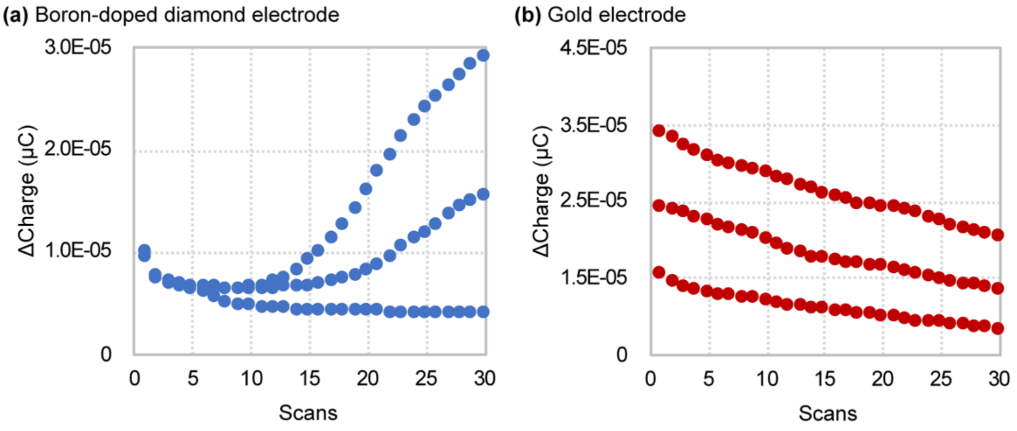

Figure S2. Electrode conditioning study on (a) boron-doped diamond and (b) gold electrodes in 1x PBS.

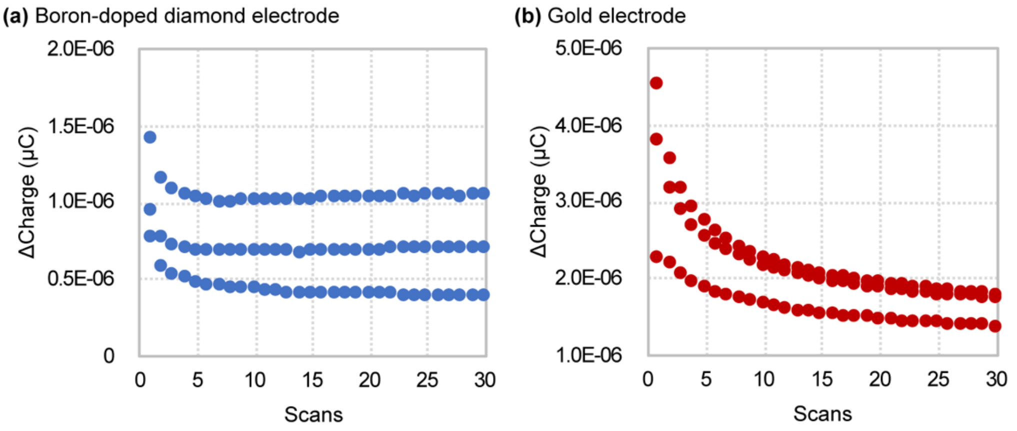

Figure S3. Surface conditioning study on (a) boron-doped diamond and (b) gold electrodes in human serum.

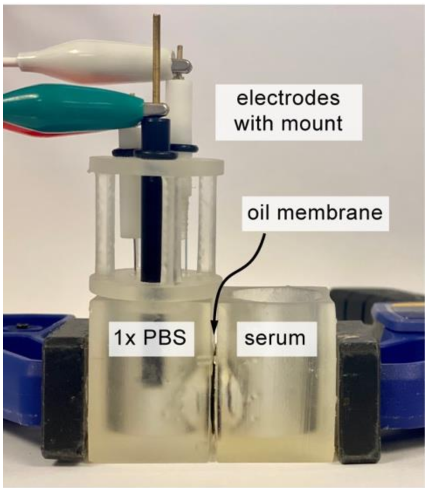

Figure S4. Labeled photograph of oil-membrane protection experiment using U-boat setup.

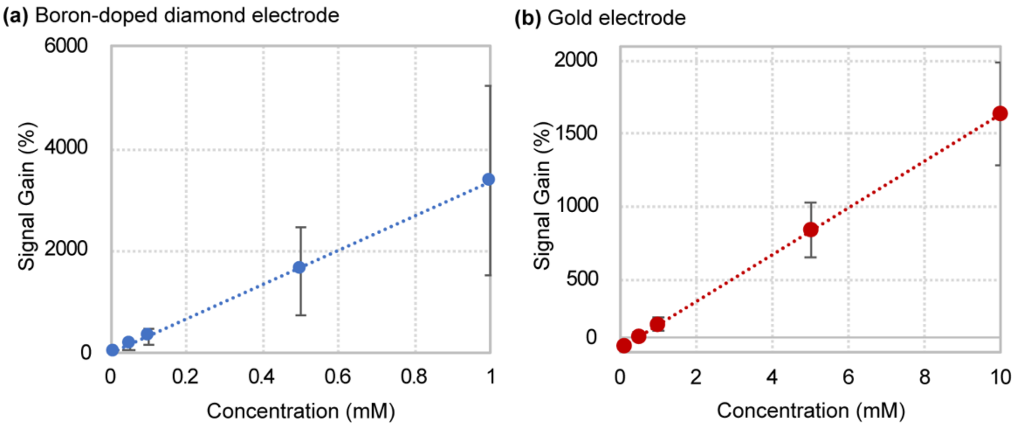

Figure S5. Hexacyanoferrate (II/III) titration curves in terms of percent signal gain for (a) boron-doped diamond and (b) gold electrodes in 1x PBS. Percent signal gain was calculated in terms of signal above baseline (0 mM redox-active species).

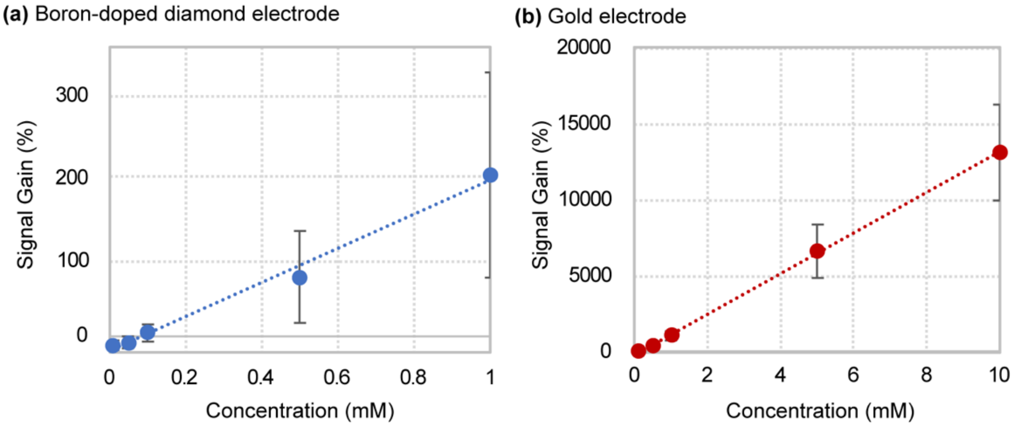

Figure S6. Hexacyanoferrate (II/III) titration curves in terms of percent signal gain for (a) boron-doped diamond and (b) gold electrodes in human serum. Percent signal gain was calculated in terms of signal above baseline (0 mM redox-active species).

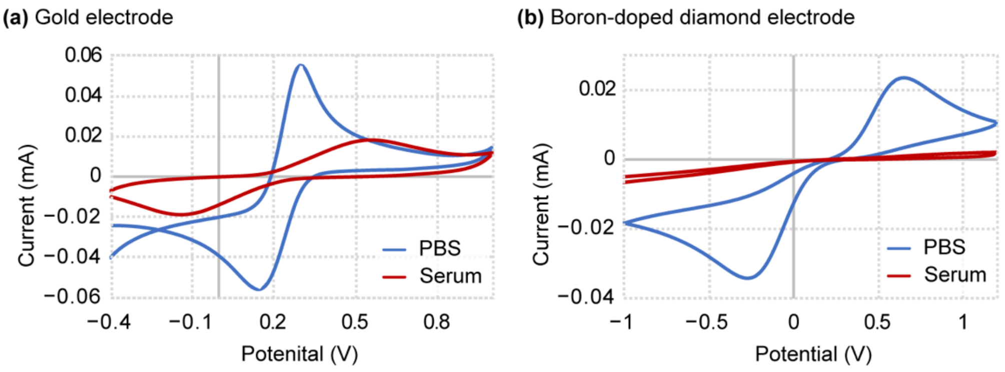

Figure S7. Hexacyanoferrate (II/III) cyclic voltammograms for (a) gold electrode and (b) boron-doped diamond electrode in 1x PBS and human serum.

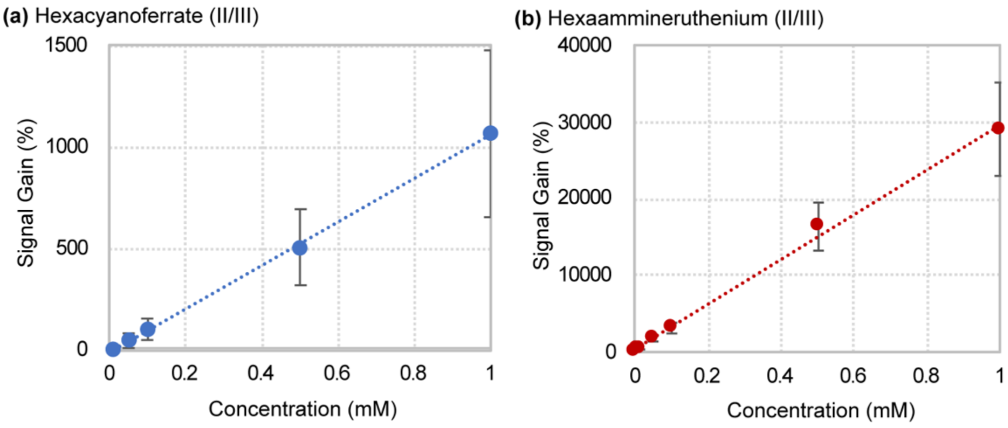

Figure S8. Oil membrane-protected boron-doped electrode titration curves in terms of percent signal gain for (a) hexacyanoferrate (II/III) and (b) hexaammineruthenium (II/III). Percent signal gain was calculated in terms of signal above baseline (0 mM redox-active species).

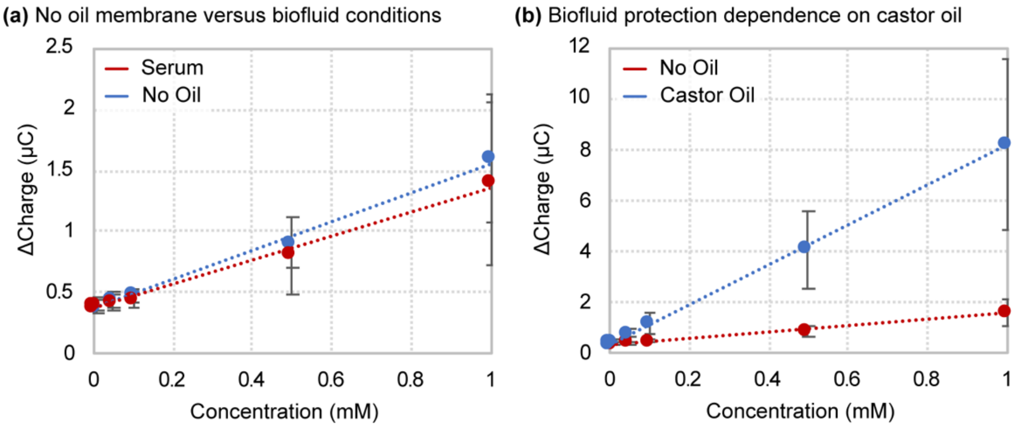

Figure S9. Hexacyanoferrate (II/III) titration and linear regression curves for (a) no-oil membrane versus biofluid conditions and (b) no-oil membrane versus castor oil membrane.

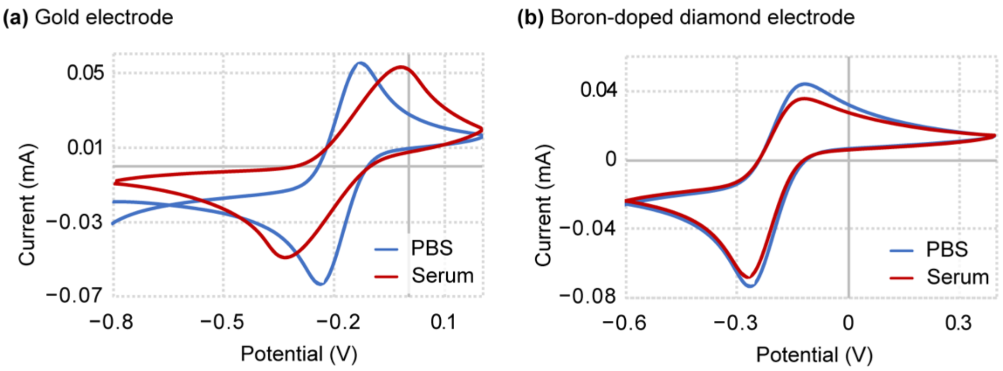

Figure S10. Hexaammineruthenium (II/III) cyclic voltammograms for (a) gold electrode and (b) boron-doped diamond electrode in 1x PBS and human serum.
